# Group A Streptococcus Vaccine Targeting the Erythrogenic Toxins SpeA and SpeB Is Safe and Immunogenic in Rabbits and Does Not Induce Antibodies Associated with Autoimmunity

**DOI:** 10.3390/vaccines11091504

**Published:** 2023-09-20

**Authors:** Matthew J. Troese, Elodie Burlet, Madeleine W. Cunningham, Kathy Alvarez, Rebecca Bentley, Nissy Thomas, Shanna Carwell, Garry L. Morefield

**Affiliations:** 1 VaxForm, LLC., Bethlehem, PA 18015, USA; 2 Department of Microbiology and Immunology, University of Oklahoma Health Sciences Center, Oklahoma City, OK 73104, USA

**Keywords:** Group A Streptococcus (GAS), *Streptococcus pyogenes*, SpeA, SpeB, vaccine, safety, autoantibodies, rheumatic heart disease (RHD), Sydenham chorea (SC), pediatric autoimmune neuropsychiatric disorder associated with group A streptococci (PANDAS)

## Abstract

Group A streptococcus (GAS) is a global pathogen associated with significant morbidity and mortality for which there is currently no licensed vaccine. Vaccine development has been slow, mostly due to safety concerns regarding streptococcal antigens associated with autoimmunity and related complications. For a GAS vaccine to be safe, it must be ensured that the antigens used in the vaccine do not elicit an antibody response that can cross-react with host tissues. In this study, we evaluated the safety of our GAS vaccine candidate called VaxiStrep in New Zealand White rabbits. VaxiStrep is a recombinant fusion protein comprised of streptococcal pyrogenic exotoxin A (SpeA) and exotoxin B (SpeB), also known as erythrogenic toxins, adsorbed to an aluminum adjuvant. The vaccine elicited a robust immune response against the two toxins in the rabbits without any adverse events or toxicity. No signs of autoimmune pathology were detected in the rabbits’ brains, hearts, and kidneys via immunohistochemistry, and serum antibodies did not cross-react with cardiac or neuronal tissue proteins associated with rheumatic heart disease or Sydenham chorea (SC). This study further confirms that VaxiStrep does not elicit autoantibodies and is safe to be tested in a first-in-human trial.

## 1. Introduction

*Streptococcus pyogenes*, known as group A streptococcus (GAS), is an exclusive human bacterial pathogen. Infections range from mild diseases, such as pharyngitis, scarlet fever, and impetigo, to severe and life-threatening diseases, which include bacteremia, puerperal sepsis, necrotizing fasciitis, and streptococcal toxic shock syndrome. Post-infection sequelae can also occur, including acute rheumatic fever (ARF), rheumatic heart disease (RHD), and acute poststreptococcal glomerulonephritis [1]. Worldwide, there are an estimated 18.1 million cases of severe disease and over 500,000 deaths annually due to *S. pyogenes* infection [2]. Although it is still currently treatable with antibiotics, resistant strains could be on the horizon as β-lactam antibiotic resistance due to mutations in the penicillin-binding protein has been reported [3]. Many low- and middle-income countries still carry the greatest risk of GAS infection and the development of RHD, especially among children [4,5]. Timely treatment with antibiotics greatly reduces the chance of severe disease but may not eliminate complications associated with infection. The development of a safe and effective vaccine is greatly needed to reduce the morbidity and mortality associated with GAS infection.

Currently, there are no licensed GAS vaccines available, and the pathway to vaccine development has been slow. A GAS vaccine using a bacterial membrane M protein led to three clinical trial participants being reported in 1969 as developing ARF [6]. Although later review of the data questioned whether the vaccine was directly responsible, at the time, the safety concern prompted the US Food and Drug Administration (FDA) in 1979 to impose a regulation that prohibited the interstate marketing of any Bacterial Vaccines and Bacterial Antigens with “No U.S. Standard of Potency” which contain group A streptococcus organisms and their derivatives [7]. It was not until 27 years later in 2006 that the FDA removed the regulation for being a “perceived impediment to the development of Group A streptococcus vaccines” [8].

The safety of a GAS vaccine is essential, as post-infection sequelae are the result of molecular mimicry of GAS antigens that cross-react with human tissues. The two studied GAS antigens responsible for autoantibodies are M proteins and group A carbohydrates (GACs) [9,10]. Historically, ARF and RHD are the most well-studied sequelae, but now neurological disorders are also recognized. Sydenham chorea (SC) and pediatric autoimmune neuropsychiatric disorder associated with streptococcal infections (PANDAS) are disorders characterized by autoantibodies targeting the basal ganglia of the brain [11,12]. In addition to autoantibodies, GAS infection can cause kidney glomerulonephritis by complement activation [13,14]. Therefore, for a GAS vaccine to be safe, it must be ensured that no antigens in the vaccine elicit adverse immune reactions.

VaxForm has developed a non-M-protein vaccine candidate comprised of a recombinant, genetically inactivated fusion protein that includes full-length streptococcal pyrogenic exotoxin A (SpeA) and mature-length streptococcal pyrogenic exotoxin B (SpeB), called SpeAB protein. SpeAB has been shown to be safely inactivated for use as an antigen [15]. Previous studies have shown that formulation with aluminum adjuvant (Alhydrogel^®^) is needed for a potent immunogenic response [16,17]. Aluminum is a standard adjuvant that has been used in vaccines since the 1930s, is approved by the FDA, and has a good safety record [18,19,20]. We call the final vaccine candidate VaxiStrep.

GAS expresses a wide variety of virulence factors that can be surface-bound and secreted [21]. We chose SpeA and SpeB to target mediators of infection and evasion of immune response that are widely conserved. SpeA is a superantigen that allows GAS to evade immune responses by interfering with adaptive immunity. SpeA can cross-link major histocompatibility complex (MHC) class II molecules, resulting in the activation of T-cells, thereby dysregulating normal immune functions [22,23]. SpeA is reported with invasive GAS disease [24,25,26]. Analyses of clinical specimens associated with disease have detected up to 90% of isolates containing the SpeA gene, although reviews of published data and sequencing data show variability between studies or geographical regions for the presence of SpeA [27,28]. Studies investigating recent outbreaks in England, including one in the fall of 2022, revealed a correlation between increases in SpeA production and invasive GAS infections [29,30]. Additional studies have shown that SpeA is an important contributor in recurrent tonsillitis in children [31]. This may be imparted by the ability of SpeA to affect T cells within the tonsils, which can further lead to B cell apoptosis and reduced antibody production [32].

SpeB is a cysteine protease that is typically secreted but can remain associated with the surface of *S. pyogenes* [33]. SpeB is secreted as an inactivated zymogen, which is processed to yield the mature protease [34,35]. SpeB has been reported to cleave over 200 substrates [36,37]. The ability of SpeB to modulate substrates such as complement and chemokines provides increased protection from host defenses [38,39]. SpeB has also been reported to affect phagocytosis [40,41]. SpeB is reported with invasive GAS disease [42,43]. Indeed, in a study of 6775 strains isolated from humans, 5711 (84.3%) expressed SpeB [44]. It is anticipated that vaccination with our SpeAB fusion protein will result in protective immunity against at least 85% of GAS isolates due to the high frequency of SpeA and SpeB. In summary, exotoxins SpeA and SpeB are highly conserved virulence factors contributing to GAS survival and pathogenesis, which makes them a prime vaccine target.

The SpeA and SpeB utilized in our SpeAB fusion protein were genetically mutated to inactivate superantigen or protease activity, respectively [15,45]. Thorough formulation studies were performed to optimize the vaccine and obtain robust immune response and stability [45]. We demonstrated that the aluminum adjuvant is needed to obtain high antibody titers and that the antibodies generated are functional and neutralize both SpeA and SpeB toxins [16]. A repeat-dose toxicity study in mice and GLP study in New Zealand White rabbits were conducted and showed no adverse events or toxicity [17]. Due to historical events related to GAS vaccine candidates potentially causing ARF and the subsequent FDA ban on GAS vaccines, this additional safety study in rabbits was performed to demonstrate that VaxiStrep does not elicit autoantibodies against the heart, kidneys, or brain.

## 2. Materials and Methods

### 2.1. VaxiStrep Vaccine Preparation

SpeA and SpeB toxin activity were inactivated by changing amino acid residue leucine 42 to Arginine (L42R) and residue cysteine 47 to serine (C47S), respectively. The inactivated SpeAB fusion protein was then cloned into a vector as previously described [15]. The protein expression and VaxiStrep preparation were described previously [16]. In brief, *E. coli* BL21 (DE3) with kanamycin resistance was cultured at 37 °C. Once the culture reached an OD600 of approximately 1.0, cells were induced with 1 mM IPTG. The temperature was reduced to 30 °C and incubated for 18 h. The cells were lysed and inclusion bodies (which contain SpeAB) were collected by tangential flow filtration (TFF). The inclusion bodies were washed with 1% sodium deoxycholate (NaDOC) and 0.5% sodium dodecyl sulfate (SDS) to remove endotoxin, then mixed with 6M urea overnight to dissolve the protein. The SpeAB protein was refolded by slowly removing urea by filtration and exchanging with 20 mM Tris with a 5 kD molecular weight cut off TFF membrane. Then 1% NaDOC was added to remove endotoxin. The NaDOC was removed with 20 mM Tris by buffer exchanging with a 30 kD TFF membrane. The SpeAB concentration was determined via total protein assay (BCA). The purification was evaluated via SDS-PAGE and antigenicity via ELISA. Once the purified SpeAB met the specifications, it was mixed with equal parts of 20 mM Tris, 20% sucrose, and 0.04% Tween 20 to obtain a final SpeAB bulk drug substance (BDS) in 20 mM Tris, 10% sucrose, and 0.02% Tween 20. The SpeAB BDS was mixed with phosphate-treated Alhydrogel^®^ (aluminum adjuvant, at 1 mg/mL) (Croda, Princeton, NJ, USA). The final vaccine formulation contained 50 µg/mL SpeAB, 1 mg/mL aluminum, in 20 mM tris, 10% sucrose, 0.02% Tween 20. Placebo control group contained 1 mg/mL aluminum, 20 mM tris, 10% sucrose, 0.02% Tween 20.

### 2.2. Wildtype SpeA and SpeB Expression

The SpeA sequence (GenBank: 216177) was cloned into a pET-24a (+) expression vector by GenScrip BioTech corporation (Piscataway, NJ, USA) using NdeI/BamHI restriction sites. The expression was similar to that described above in the VaxiStrep vaccine preparation section. In short, *E. coli* BL21 (DE3) with kanamycin resistance was cultured at 37 °C. Once the culture reached an OD600 of approximately 0.5–0.9, the cells were induced with 20 mM IPTG (Promega, Madison, WI, USA). The temperature was reduced to 30 °C and incubated for 18 h. The following day, the *E. coli* pellet was collected, washed and frozen at −20 °C. The frozen cells were thawed, resuspended in water, vortexed, and treated with 0.01% universal nuclease (Thermo Fisher, Waltham, MA, USA). The lysed *E. coli* cell debris was removed by centrifugation and the supernatant (containing soluble SpeA in PBS) was collected. To remove endotoxin, a Triton-X-114 phase separation method was performed [46]. In short, 0.5% Triton-X-114 was added to the SpeA protein solution and mixed at 4 °C for 30 min. Then the sample was placed at 37 °C for 10 min, causing the Triton-X-114 to precipitate out of solution with the endotoxin. The sample was centrifuged to pellet the Triton-X-114 with endotoxin, while the supernatant contained SpeA. This process was repeated 3 times to remove endotoxin. The identity of the wildtype SpeA protein was evaluated via SDS-PAGE and silver stain, which identified a protein of approximately 26 kDa.

The SpeB sequence (GenBank: M86905.1) corresponding to the mature protein (amino acids 146-398) [34] was cloned and expressed as described for wildtype SpeA. After overnight expression, the *E. coli* pellet was collected, washed with water, and then mixed with 0.5% EDTA, 0.5% SDS, 1% NaDOC, and 0.1% Triton-X-100 for 1 h at room temperature. Then universal nuclease (Thermo Fisher, Waltham, MA, USA) was added to a final concentration of 0.01% and mixed for 30 min. Insoluble inclusion bodies were washed with water repeatedly until detergents could no longer be detected via OD215 readings. The inclusion bodies containing SpeB were solubilized in 3M Urea/3M Guanidine-HCl. SpeB was refolded by buffer exchange and concentrated using 3 kDa spin filters (MilliporeSigma, Burlington, MA, USA). The identity of the wildtype SpeB protein was evaluated using SDS-PAGE and silver stain, which identified a protein of approximately 27 kDa.

### 2.3. Rabbit Studies

Safety and immunogenicity studies were performed in New Zealand White rabbits (Envigo, Madison, WI, USA). Administration of the test articles, in-life monitoring, and necropsy (including organ harvest and serum collection) were conducted by Frontage Laboratories, Inc. (Concord, OH, USA). The age of animals at the time of dosing was 4–5 months. All animals were experimentally naïve (10 males and 10 females). The placebo and VaxiStrep test articles were provided by VaxForm as ready-to-inject formulations. All administrations were given via intramuscular injection (IM) in the right hindlimb of the rabbits. Placebo or VaxiStrep were administered to 10 rabbits each (5 male and 5 female), on days 1 and 29 at 0 or 25 µg/animal/dose, respectively, at a dose volume of 0.5 mL/animal to mimic the human dose. Blood samples for immunogenicity testing were collected from all animals during the pre-dose period (day—1), and on days 15, 22, 29, and 33. The animals were terminated on day 33. The heart, kidneys, and brain were collected from all terminated animals as whole organs and preserved in 10% neutral-buffered formalin for immunohistochemistry.

### 2.4. Serology Analysis

Anti-SpeAB IgG ELISAs were performed on collected rabbit serum by VaxForm. ELISA plates (Greiner Bio-One, Monroe, NC, USA) were coated with 5 µg/mL SpeAB protein in PBS for 1 h at 37 °C, washed 3 times with PBS-Tween 20 (0.05%), and blocked with 1% BSA in PBS (Thermo Scientific, Waltham, MA, USA) for 1 h at 37 °C. Rabbit serum from individual animals was serially diluted two-fold in 1% BSA in PBS-Tween 20 (0.05%) and incubated for 2 h at 37 °C, then washed 3 times with PBS-Tween 20 (0.05%). Anti-rabbit IgG-HRP antibody (Abcam, Waltham, MA, USA) diluted to 1:200,000 in 1% BSA in PBS-Tween 20 (0.05%) was incubated for 1 h at 37 °C. The plate was washed 3 times with PBS-Tween 20 (0.05%). TMB substrate (Sera Care, Milford, MA, USA) was added for 30 min, after which 3M sulfuric acid was added to stop the reaction. The plate was read at 450 nm (Molecular Devices M2e plate reader). To calculate titers, a cutoff of 3 times the background was used in each ELISA. The samples were diluted such that one dilution was above the cutoff and the next dilution was below the cutoff. Anti-SpeA and anti-SpeB ELISA were performed as described above with the following exceptions. The plates were coated with wildtype 5 µg/mL SpeA or wildtype 1 µg/mL SpeB. For the anti-SpeB ELISA, serum samples were diluted in 1% BSA in PBS. Excel slope and intercept functions were used to interpolate the dilution value (titer) at the cutoff value.

### 2.5. Immunohistochemistry

Immunohistochemistry procedures were performed as previously described [47]. In short, tissues were blocked overnight in 1% goat serum in Power Block (BioGenex, Fremont, CA, USA). Biotinylated goat anti-rabbit IgG (10 µg/mL) (Sigma-Aldrich, St. Louis, MO, USA) or-PBS negative control as well as positive control rabbit anti-cardiac myosin sera (Cunningham laboratory) at 1:2000 dilution in PBS were incubated with tissues, followed by streptavidin incubation and Fast Red detection with a counterstain of Mayer’s hematoxylin. The positive control sera were produced via the hyperimmunization of New Zealand White rabbits with purified human cardiac myosin prepared and analyzed using standard methods in the Cunningham laboratory.

### 2.6. Lysoganglioside-GM1, Tubulin, D1R, and D2R ELISAs to Detect the Presence of Anti-Neuronal Autoantibodies in Rabbit Sera Immunized with SpeAB

ELISAs were performed using 4 different neuronal antigens to detect autoantibodies in rabbit serum. Ninety-six well plates were coated overnight in 0.015 M carbonate/0.03 M bicarbonate (pH 9.6) buffer with each of the 4 antigens (Lysoganglioside, tubulin, human dopamine D1 receptor [D1R] antigen, and human dopamine D2 receptor [D2R]) as described previously [48]. ELISAs were then performed similar to what was described [49]. In short, washes were performed with PBS containing 0.05% Tween (PBS-Tween) five times and then blocked for 1 h. The wells were washed and sera from immunized or placebo rabbits were diluted and incubated overnight. After five washes, the samples were incubated with alkaline-phosphatase-labeled goat anti-rabbit IgG (Sigma-Aldrich, St. Louis, MO, USA). Then substrate and p-nitrophenyl phosphate were added. The optical density (OD) was measured at 405 nm. The results are expressed as the mean of triplicate wells. All ELISAs were validated with known positive and negative serum controls for each assay to maintain a standardized assay.

### 2.7. Calcium Calmodulin Kinase II (CaMKII) Assay to Detect the Presence of Human SKNSH Neuronal Cell Signaling by Rabbit Sera

The assay was performed as previously described [49]. In short, SKNSH human neuroblastoma cells were placed overnight in complete DMEM. The next day, the cells were serum-starved and treated with sera (1/100 dilution) separately from placebo and immunized rabbits. The cells were harvested, centrifuged, and solubilized. The enzymatic activity was measured using the CaMKII assay system (Promega, Madison, WI, USA) per manufacturer instructions using ATP [γ-32P]. The samples were spotted onto membranes. A scintillation counter (Beckman Coulter, Indianapolis, IN, USA) was used to measure radioactivity on the membrane. The protein concentration of each sample was used to standardize the CaMKII enzyme activity. The results were averaged from triplicate assays. CaMKII activity was reported as the percent above the basal level. Each assay was validated and standardized with known positive and negative serum controls.

### 2.8. Human Cardiac Myosin (HCM) ELISA to Detect the Presence of Autoantibodies against the Heart in Rabbit Sera Immunized with SpeAB

HCM was purified from human myocardium. HCM (10 µg/mL) was coated onto a 96-well ELISA plate. The coating and ELISA procedures were the same as those described above for Lysoganglioside-GM1, tubulin, D1R and D2R ELISAs.

### 2.9. Protein Kinase A (PKA) Cardiomyocyte Signaling Assay

H9c2 primary heart cells (ATCC, Manassas, VA) were plated in 6-well plates at 2.5 million cells/well overnight in complete DMEM media (Thermo Fisher, Waltham, MA, USA), at 37 °C with 5% CO_2_. An assay was performed as previously described [50]. In short, the following day, rabbit serum (1/100 dilution) from placebo and immunized groups were incubated separately with heart cell line in a final volume of 2 mL of medium. The cells were mechanically dislodged, centrifuged, and solubilized in extraction buffer. Protein kinase A (PKA) activity was measured with a SignaTECT PKA assay system (Promega, Madison, WI) per manufacturer’s instructions. The PKA activity is reported as a percent above the basal level.

### 2.10. Statistical Analysis

Unpaired *t*-tests comparing placebo vs. VaxiStrep treatment were performed using the GraphPad *T* test calculator website: https://www.graphpad.com/quickcalcs/ttest1.cfm (accessed on 12 September 2023).

## 3. Results

### 3.1. Two Dose Study in New Zealand White Rabbits

**Figure 1:**
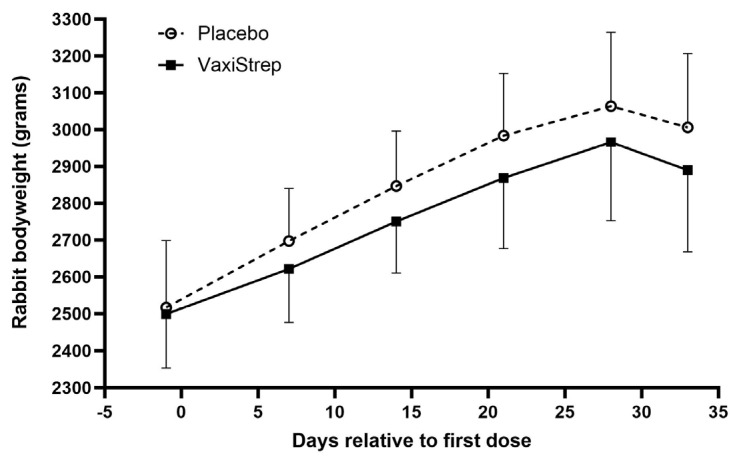
Mean bodyweights of New Zealand White rabbits that received either placebo or VaxiStrep. A total of 10 rabbits (5 male and 5 female) received either placebo or VaxiStrep on day 1 and day 29. Days are relative to the first dose of placebo and VaxiStrep. Bodyweights were taken on days—1 (pre-dose), 7, 14, 21, and 28, and a terminal bodyweight was taken on day 33. The average and standard deviation are shown for each group (male and female). No significant statistical difference was observed between placebo and VaxiStrep groups at all time points. For days—1, 7, 14, 21, 28, and 33, unpaired *t*-test *p*-values were 0.8156, 0.2519, 0.1558, 0.1713, 0.3071, and 0.2354.

To evaluate the safety of our GAS vaccine, the New Zealand White rabbits received two doses of VaxiStrep (25 µg/dose) to imitate the dose administration schedule and amounts that would be given to humans. Five male and five female rabbits were administered either placebo control (aluminum only) or VaxiStrep (SpeAB/aluminum) on days 1 and 29 via intramuscular injection. The animals were terminated on day 33. All animals survived throughout the study. During the dosing period, no VaxiStrep-related adverse findings were noted. The bodyweights were taken throughout the study (days relative to first dose) on days—1, 7, 14, 21, and 28, and a terminal weight was taken on day 33 (Figure 1). No significant change in body weight was noted in animals administered VaxiStrep compared to the placebo controls.

### 3.2. Organ Weights

Organ weight changes can be used to evaluate the toxicity of administered compounds [51]. Since GAS infection has been linked to heart and kidney disease, the heart and kidneys were collected from all terminated rabbits. All organs appeared normal at the time of harvest. The heart and kidneys were weighed and no VaxiStrep-related significant change in organ weight was observed compared to the placebo controls (Table 1).

### 3.3. Immunogenicity of VaxiStrep

**Figure 2:**
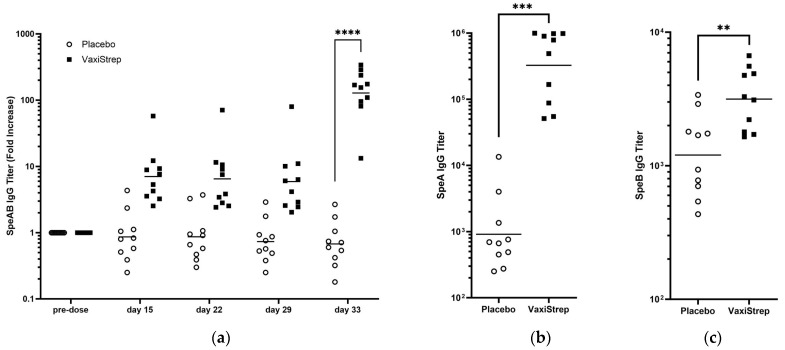
Immunogenicity of VaxiStrep in New Zealand White rabbits and individual SpeA and SpeB titers. A total of 10 rabbits (5 male and 5 female) received either placebo or VaxiStrep on day 1 and day 29. (**a**) Blood samples were collected on days—1 (pre-dose), 15, 22, 29, and 33, and individual serum IgG SpeAB titers were determined. The SpeAB serum titers are presented as a fold increase over pre-dose for days 15, 22, 29, and 33; unpaired *t*-test *p*-values between placebo and VaxiStrep groups were 0.0665, 0.1059, 0.1343, and <0.0001, respectively. (**b**) Individual day 33 serum IgG SpeA titers. (**c**) Individual day 33 serum IgG SpeB titers. Unpaired *t*-test values were 0.0007 and 0.0053 for SpeA and SpeB, respectively, between Placebo and VaxiStrep. The geometric mean is shown by a horizontal line. (**: *p* < 0.01, ***: *p* < 0.001, ****: *p* ≤ 0.0001).

To determine the titer of antibody response that was generated to VaxiStrep, individual rabbit SpeAB-specific IgG titers were evaluated via ELISA on day 33 serum (Figure 2a). The SpeAB serum titers are presented as a fold-increase over pre-dose (day—1) samples. A significant increase in serum IgG titer was observed for VaxiStrep compared to placebo controls. Individual IgG titers from day 33 serum for SpeA (Figure 2b) and SpeB (Figure 2c) were evaluated using wildtype proteins.

### 3.4. Immunohistochemistry of Rabbit Brain, Heart, and Kidney Tissue

**Figure 3:**
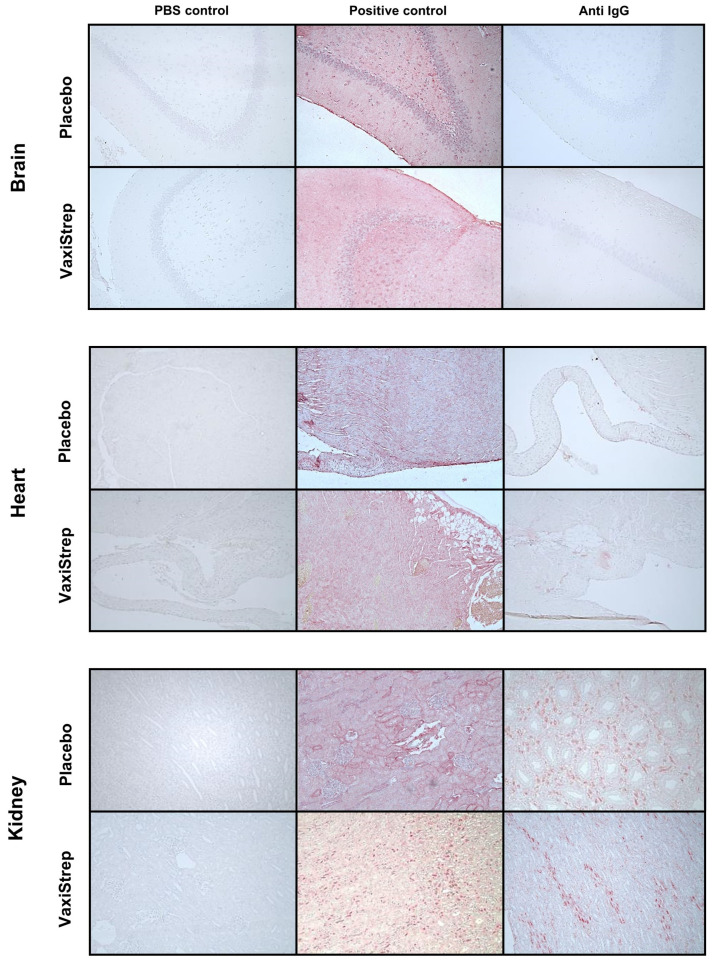
Immunohistochemistry staining for IgG in rabbit brain, heart, and kidney tissue. From each placebo and VaxiStrep group, a randomized subset of 5 rabbits (2 males and 3 females) were analyzed for IgG deposits in tissues. Biotinylated goat anti-rabbit IgG Ab was followed by alkaline phosphatase-conjugated streptavidin with Fast Red substrate to detect antibody binding, with a counterstain of Mayer’s hematoxylin. Positive control was rabbit anti-cardiac myosin serum (2000×). Representative images are shown.

Having confirmed a strong anti-SpeAB IgG response was generated, we next sought to evaluate rabbit brain, heart, and kidney tissues to determine if IgG might have bound tissues due to the cross-reactivity of autoantibodies generated by the vaccine or if there were immune complex deposits. Autoantibodies against GAS antigens can react with heart and brain tissue, and immune complexes can form in the kidney, causing glomerulonephritis [52,53,54]. The tissues were processed in formalin for immunohistochemistry. Antibody binding was detected via anti-IgG conjugated to alkaline phosphatase and developed with Fast Red substrate. The representative images are shown in Figure 3. A red staining color on the tissues indicated a positive response as seen in the positive control anti-HCM-treated tissue sections, which are used for comparison with the immunized and PBS negative controls. No positive IgG staining was observed in the rabbit heart and brain tissues of the placebo and VaxiStrep groups. There was no apparent difference observed in the kidney tissues in the PBS negative control, placebo, and immunized VaxiStrep rabbits. Some light staining could be seen in glomeruli, but this was observed for placebo and VaxiStrep-immunized rabbits and appeared the same at all dilutions. The light staining occurred at specific locations and was not equivalent to the dispersed red staining seen with the positive control. See the Figure 3 comparison, where there is no difference in the placebo and VaxiStrep immunized rabbit kidneys.

### 3.5. Evaluation of Autoantibodies (IgG) to Heart Antigens

We next sought to study the immunized rabbit serum in assays to evaluate reactivity with human heart antigens. Placebo and VaxiStrep sera were tested for reactivity with human cardiac myosin (HCM). In addition, the sera were tested for the activation of protein kinase A (PKA) cell signaling in ATCC H9c2 heart cells in culture. PKA signaling reflects the cross-reactivity of the autoantibodies against HCM with the beta adrenergic receptor as previously described [50]. Sera from Placebo and VaxiStrep animals that were collected before treatment (pre-dose, day—1) and after treatment (day 33) were tested. The HCM titers as well as the PKA assay values from placebo and VaxiStrep sera (pre-dose and day 33 serum) are listed in Table 2. The pre-dose values serve as a frame of reference for comparison. When day 33 placebo serum assay values were compared to day 33 VaxiStrep serum values, no statistical significance was observed for either assay.

### 3.6. Evaluation of Autoantibodies (IgG) to Brain Antigens

Antibodies to GAS that target GAC or streptococcal M protein peptides can cross-react with human brain tissues [9]. Therefore, rabbit serum IgG was tested in a group of anti-neuronal autoantibody assays that have been used to evaluate neurological disorders associated with human GAS infection [49,55]. The antigens included Dopamine D1 receptor (D1R), Dopamine D2 receptor (D2R), Tubulin, and Lysoganglioside-GM1 (Lyso-GM1) as well as calcium/calmodiulin-dependent protein kinase II (CaMKII) used to measure neuronal cell CaMKII activation. Sera from Placebo and VaxiStrep animals that were collected before treatment (pre-dose, day—1) and after treatment (day 33) were tested (Table 3). The pre-dose values serve as a frame of reference for comparison. When day 33 placebo serum assay values were compared to day 33 VaxiStrep serum assay values, no statistical significance was observed for any of the assays.

## 4. Discussion

The path to developing a GAS vaccine has been hampered by several obstacles. These include a lack of animal models to effectively study this exclusively human pathogen, the diversity of bacterial strains, and most significantly, the risk of GAS antigens causing autoimmunity. The challenge of M protein vaccine candidates is targeting conserved regions of the diverse M protein sequences [56]. To provide optimal protection, various combinations of GAS virulence factors are typically used in vaccine formulations. Only a few of these vaccine candidates have undergone Phase I trials, with many more in preclinical development [57,58].

VaxForm has developed a GAS vaccine candidate using antigens expressed by over 85% of serotypes that do not present a risk of autoimmunity. The vaccine candidate, called VaxiStrep, was shown to be stable for at least 2 years at 4 °C and can elicit functional anti-SpeA and anti-SpeB antibodies that can neutralize native GAS toxins [16]. Previous studies conducted in mice and rabbits showed no evidence of systemic toxicity caused by the vaccine [17]. However, due to the potential severity of autoimmunity complications and additional FDA scrutiny due to historical safety issues with GAS vaccine testing, this study was conducted to further demonstrate the safety of VaxiStrep and ensure that it did not generate cross-reactive autoantibodies. New Zealand White rabbits were administered two doses of VaxiStrep (25 µg/dose) by intramuscular injection 2 weeks apart.

During this study, no VaxiStrep-related adverse findings were observed. There was no mortality or adverse reported site injection reactions in Placebo or VaxiStrep groups. Overall, the results demonstrate that the vaccine was well tolerated both locally and systemically in rabbits. The high antigen-specific titers detected two weeks after the second dose validates we can confidently test for the presence of an autoantibody response to ensure the safety of our vaccine, as it is the molecular mimicry of GAS antigens that results in the production of autoantibodies that are responsible for GAS post-infection sequelae. Day 33 serum was analyzed for individual anti-SpeA and anti-SpeB IgG antibodies to wildtype proteins as well as fusion protein SpeAB titers. Although the anti-SpeA titers were higher than anti-SpeB, we believe that this may be due to differences in ELISA optimization. We have previously demonstrated that immunization in mice with SpeAB was able to generate antibodies capable of neutralizing wildtype SpeA and SpeB activity [16], suggesting that the fusion of the inactivated proteins still maintains similar structural homology to wildtype native proteins.

The three post-streptococcal infection sequelae that were investigated in this vaccine safety study included heart, kidney, and brain pathologies. Vaccinated and placebo rabbit sera were tested for IgG deposits in all three tissues to rule out vaccine-induced pathologies. When staining for IgG deposits in the kidneys, there was a low background detection of IgG in both groups but no differences between VaxiStrep-treated rabbits compared to placebo tissues. While animal models that mimic human GAS infection are still a challenge, rabbit models have shown promise in eliciting post-streptococcus glomerulonephritis [53]. Previous studies have shown mice and rabbit autoantibodies directly reacting to human renal glomeruli tissues [59,60,61]. However, most of the literature reports that kidney damage is not a direct result of autoantibodies, but instead immune complex deposits and C3a complement activation [13,62,63]. SpeB has been reported as a possible antigen associated with poststreptococcus glomerulonephritis [64]. In this study, we saw no increased staining in VaxiStrep-treated rabbit kidney tissues compared to placebo, indicating no antibodies bound to kidney tissues and that no IgG-SpeAB immune complexes were deposited within the kidneys.

The two most studied GAS antigens responsible for autoantibodies are M proteins and group A carbohydrate (GAC). Streptococcus M proteins are a surface-expressed protein that extends from the cell wall and contains conserved and variable regions, which have been extensively analyzed for structure and amino acid conservation [65]. The M proteins form a α-helical coiled-coil structure [66], which serve as the basis for cross-reactivity to human heart tissues. The S2 region of cardiac myosin is the target of autoimmune responses against the heart [50]. Additionally, anti-myosin antibodies can target the beta-adrenergic receptor, which induces cAMP-dependent protein kinase A (PKA) activity and cell signaling activity in heart cells [47,50]. In this study, we evaluated rabbit serum for cross-reactivity with cardiac myosin and activation of PKA signaling. The results indicate that there was no statistical significance between placebo and VaxiStrep day 33 serum, and no significantly elevated responses were detected for either HCM or PKA. This evidence demonstrates that VaxiStrep did not induce serum autoantibodies to heart antigens, thereby excluding the chance of inducing ARF and RHD.

ARF and RHD have been the focus of historical GAS post-infection sequelae; however, more recently it has been shown that infection can also result in neurological disorders. Sydenham chorea (SC) is a movement disorder after GAS infection, and the neurologic manifestation results in random, involuntary, and uncoordinated movements [11]. Another GAS neurological disorder is PANDAS, used to describe acute-onset obsessive-compulsive disorders and/or tics along with psychological symptoms in children with prior Streptococcus infection [12]. Although the identification of specific markers to diagnostically distinguish Sydenham chorea and PANDAS is progressing [67,68], both disorders are characterized by autoantibodies targeting the basal ganglia of the brain. To determine if SpeAB antibodies cross-react with brain antigens, rabbit serum was evaluated in a panel of five assays used to identify PANDAS [55].

The basal ganglia antigens of interest, which are targeted by autoantibodies after GAS infection include the dopamine receptors D1R and D2R [48,69]. Antibodies to D1R and D2R result in excess dopamine release that leads to the involuntary movements associated with Sydenham chorea and PANDAS. Another autoantigen is β-tubulin, which is an abundant intracellular protein in the brain [70]. Although there is no direct pathology associated with anti-tubulin antibodies, they provide a diagnostic indication of GAS infection and are the same antibodies that cross-react with GAC, and they also bind the antigen Lyso-ganglioside-GM1 [71]. Gangliosides are a major component of neuronal cells and modulate cell growth and signal transduction [72]. The detection of these autoantibodies to these neuronal autoantigens was evaluated via ELISA titers. The autoantibody-mediated neuronal cell signaling was evaluated for CaMKII activation [73]. In the event that antibodies cannot be detected directly via ELISA, the CaMKII assay provides a functional signaling endpoint by measuring human neuronal cell stimulation. This occurs through the activation of calcium-calmodulin-dependent protein kinase II (CaMKII) and an increase in tyrosine hydroxylase in dopaminergic neurons, which leads to elevated dopamine release. For D1R, D2R, tubulin, lysoganglioside-GM1, and CaMKII, no significant differences were detected between day 33 placebo sera and day 33 VaxiStrep sera. These assays demonstrate that VaxiStrep immune serum did not induce elevated autoantibodies to brain antigens, thereby excluding the chance of inducing basal ganglia encephalitis.

*Streptococcus pyogenes* is an exclusive human pathogen. This makes the use of any animal model a challenge and potentially limits how it can directly correlate with human disease and pathology. For that reason, different animal models are used to study different pathologies [74].

Over the years, the animal models of ARF and RHD have included the use of guinea pigs, rabbits, pigs, sheep, goats, cattle, cats, dogs, and non-human primates [75]. The use of rabbits in evaluating RHD autoimmune antibodies has seen various results in different studies [76], but autoantibodies have been observed in rabbits from antigen administration [54,77,78]. For this study, New Zealand White rabbits were used for their versatility as models for vaccinology, toxicology, and pathologies similar to humans. We chose to test our vaccine candidate in rabbits as they are routinely used for toxicology safety assessments. The rabbits were also administered vaccine doses equivalent to what humans would receive. Since autoantibodies have been detected in rabbits, we evaluated the vaccinated rabbit tissues for IgG deposits. In more recent years, the Lewis rat model has gained more recognition for cardiac and neuronal autoantibody modeling [79].

To substantiate the negative autoantibody immunohistochemistry results, rabbit serum was further evaluated in ELISAs and cell signaling assays for cardiac and neuronal cross-reactivity. The use of vaccinated animal sera to screen for potential autoantibodies in human tissues has been performed in other studies. Sera from mice and rabbits vaccinated with M proteins have shown reactivity to human heart lysates and human brain antigens, respectively [80,81]. Also, a multivalent vaccine containing amino-terminal M protein fragments was administered to rabbits, and the serum was evaluated for cross-reactivity to human brain, kidney, and heart tissues to demonstrate the safety of the vaccine [82]. Human clinical trials have also evaluated sera from patients for cross-reactivity to human tissues samples to look for the presence of autoantibodies [83]. These data suggest that the use of human antigens and tissues have been successfully used to evaluate the presence of autoantibodies.

One limitation of this study is that cross-reactive T-cells were not evaluated. Studies have shown that T-cells also contribute to the pathology of RHD [84,85]. The specific events required to initiate RHD after GAS infection are still unclear [86]. It has been suggested that RHD is initiated by autoantibody responses [87]. Regardless of how RHD is initiated, the presence of autoantibodies in humans is associated with an increased risk of ARF and RHD [88,89].

Preclinical safety data are a prerequisite before any vaccine is administered to humans. Trying to model GAS infection and pathology is notably more challenging with the lack of good animal models that fully encompass more aspects of human disease. This makes it challenging to fully predict immune responses and whether differences in antibody and/or T-cell epitopes between humans and animals could potentially lead to different outcomes. The lack of animal models to support GAS infection also makes it difficult to fully evaluate vaccine efficacy. Demonstrating a vaccine provides good protection in animal models may not correlate to humans if the mechanisms of colonization and infection are different. This necessitates that to fully evaluate the efficacy of a vaccine candidate, it will need to be evaluated in human clinical trials.

Due to the potential of developing autoantibodies from GAS, safety testing must be performed before any GAS vaccine enters clinical trials in humans. The development of RHD and the more recently recognized basal ganglia encephalitis present a serious concern. Although both conditions are known to develop only from cross-reactivity from M proteins and GAC, we evaluated VaxiStrep for the same risk. This study further confirms the safety of the VaxiStrep vaccine by demonstrating the antigen does not elicit any autoantibodies that react with the heart and neuronal tissues or antigens tested and do not signal PKA or CaMKII in heart or neuronal cells, respectively. The safety data for VaxiStrep, combined with the high percentage of protection it would provide from invasive GAS strains, makes this vaccine an ideal candidate for future clinical trials.

## 5. Conclusions

Due to the historical safety issues of GAS vaccines to potentially induce autoimmunity, this study was conducted to ensure the safety of our vaccine candidate (VaxiStrep). Intramuscular injection of our recombinant SpeAB fusion protein with aluminum adjuvant generated a robust antibody response in rabbits. No deposition of IgG could be detected in rabbits’ hearts, brains, and kidneys. Rabbit serum was evaluated for autoantibodies to cardiac or neuronal proteins associated with RHD and Sydenham chorea. This preclinical study shows no indications that autoantibodies are generated from VaxiStrep and demonstrates its readiness for first-in-human clinical trials.

## Figures and Tables

**Table 1:** Terminal body, heart, and kidneys weights.

Treatment	Sex	Day 33 Terminal Body Weight (g)	Heart Weight (g)	Heart to Body Weight (%)	Kidney Weight (g)	Kidneys to Body Weight (%)
Placebo	Female	3234.6	5.860	0.181	17.245	0.533
		3169.9	6.306	0.199	18.640	0.588
		3031.2	8.262	0.273	16.470	0.543
		3137.5	8.048	0.257	16.016	0.510
		3243.1	6.972	0.215	15.825	0.488
	Male	2679.2	6.503	0.243	13.678	0.511
		2954.2	6.530	0.221	18.279	0.619
		2796.4	7.492	0.268	15.831	0.566
		3040.9	7.675	0.252	17.997	0.592
		2772.1	7.347	0.265	18.388	0.663
	AVG	3005.9	7.100	0.237	16.837	0.561
	STD	200.7	0.793	0.032	1.566	0.055
VaxiStrep	Female	2740.2	6.977	0.255	16.316	0.595
		3176.2	7.531	0.237	20.855	0.657
		2808.6	9.870	0.351	15.874	0.565
		3189.7	6.861	0.215	16.488	0.517
		3064.5	6.846	0.223	16.155	0.527
	Male	2557.3	5.758	0.225	13.173	0.515
		2588.3	5.883	0.227	14.043	0.543
		2942.5	6.775	0.230	15.646	0.532
		2962.4	6.978	0.236	17.579	0.593
		2869.2	6.812	0.237	16.133	0.562
	AVG	2889.9	7.029	0.244	16.226	0.561
	STD	221.5	1.127	0.039	2.052	0.044
*p*-value		0.2340	0.8735	0.7019	0.4640	0.9753

Average (AVG) and standard deviation (STD). Unpaired *t*-test indicates no statistical difference was observed between placebo and VaxiStrep groups for body, heart, or kidney weight.

**Table 2:** Cross-reactivity of placebo and VaxiStrep serum to heart antigens.

		Pre-Dose Control Serum		Day 33 Serum	
Treatment	Sex	HCM Titer	PKA	HCM Titer	PKA
Placebo	Female	800	0	400	0
		400	0	400	3.17
		400	10.20	400	0
		400	3.23	400	0
		400	0	400	2.42
	Male	400	0.46	800	0
		200	0.46	400	0
		400	0	400	0
		400	15.44	400	29.63
		400	0	400	1.57
	AVG	420	2.98	440	3.68
VaxiStrep	Female	800	1.48	800	2.87
		800	8.49	400	2.35
		1600	10.24	800	7.27
		800	0	800	6.73
		800	8.98	400	0
	Male	400	0	400	0
		800	0	400	0
		400	7.12	400	0
		800	0	800	0
		400	5.75	800	0
	AVG	760	4.21	600	1.92
			*p*-value	0.0544	0.5714

Elevated human response criteria for heart cross-reactivity: human cardiac myosin (HCM): normal titer range 100–800 (Positive ≥ 1600). HCM is listed as titers. PKA: 30% above basal levels. PKA values are reported as % above basal rate. High (HC) and low controls (LCs) were run as an internal standard; HCM: HC 12,800, LC 800. PKA: HC 132, LC 0.015. Unpaired *t*-test indicated no statistical difference between placebo and VaxiStrep on day 33 sera for either assay.

**Table 3:** Cross-reactivity of placebo and VaxiStrep sera to brain antigens.

		Pre-Dose Control Serum					Day 33 Serum				
Treatment	Sex	D1R	D2R	Tubulin	Lyso-GM1	CaMKII	D1R	D2R	Tubulin	Lyso-GM1	CaMKII
Placebo	Female	1600	800	400	1600	125	800	800	400	1600	133
		800	800	400	400	121	800	800	400	400	87
		400	400	3200	200	126	800	800	1600	800	112
		400	400	800	200	100	400	400	400	200	108
		400	400	200	200	120	400	400	400	200	109
	Male	400	1600	800	200	114	800	800	800	100	97
		800	800	200	100	131	400	400	400	100	113
		800	800	100	400	136	800	400	200	400	146
		800	800	800	100	146	1600	800	800	200	150
		1600	800	400	100	130	800	800	800	100	142
	AVG	800	760	730	350	125	760	640	620	410	120
VaxiStrep	Female	800	400	400	200	125	800	800	800	400	113
		800	400	200	100	141	400	400	400	200	115
		1600	800	3200	100	126	1600	800	1600	100	137
		400	800	400	200	126	800	800	200	400	143
		1600	800	400	100	120	1600	800	800	200	108
	Male	400	400	200	100	134	400	200	200	100	134
		800	400	400	200	155	800	400	400	400	159
		400	400	400	200	127	800	800	800	200	104
		400	400	200	200	128	200	400	800	400	123
		400	400	200	400	117	800	800	400	400	122
	AVG	760	520	600	180	130	820	620	640	280	126
					*p*-value		0.7486	0.8437	0.9148	0.4108	0.4952

Positive human serum response criteria for brain cross-reactivity: D1R: ≥4000. D2R: ≥16,000. Tubulin: ≥2000. lyso-ganglioside-GM1: ≥640. D1R, D2R, Tubulin, and lyso-ganglioside-GM1 are listed as titers. CaMKII: ≥130% above basal. CAMKII values are reported as a % increase above basal levels. High (HCs) and low controls (LCs) were run as an internal standard; D1R: HC 1000, LC 800, D2R: HC 16,000, LC 800, Tubulin: HC 16,000, LC 800, Lyso-GM1: HC 800, LC 200, CaMKII: HC 163, LC 100. Unpaired *t*-test indicated no statistical difference between placebo and VaxiStrep groups on day 33 sera for any assay.

## Data Availability

Not applicable.
